# Evaluation of different chrominance models in the detection and reconstruction of faces and hands using the growing neural gas network

**DOI:** 10.1007/s10044-019-00819-x

**Published:** 2019-04-08

**Authors:** Anastassia Angelopoulou, Jose Garcia-Rodriguez, Sergio Orts-Escolano, Epaminondas Kapetanios, Xing Liang, Bencie Woll, Alexandra Psarrou

**Affiliations:** 1 School of Computer Science and Engineering, University of Westminster, 115 New Cavendish Street, London W1W 6UW, UK; 2 Department of Computing Technology, University of Alicante, Po Box 99, 03080 Alicante, Spain; 3 Deafness Cognition and Language Research Centre, University College London, 49 Gordon Square, London WC1H 0PD, UK

**Keywords:** Expectation maximisation (EM) algorithm, Colour models, Self-organising networks, Shape modelling

## Abstract

Physical traits such as the shape of the hand and face can be used for human recognition and identification in video surveillance systems and in biometric authentication smart card systems, as well as in personal health care. However, the accuracy of such systems suffers from illumination changes, unpredictability, and variability in appearance (e.g. occluded faces or hands, cluttered backgrounds, etc.). This work evaluates different statistical and chrominance models in different environments with increasingly cluttered backgrounds where changes in lighting are common and with no occlusions applied, in order to get a reliable neural network reconstruction of faces and hands, without taking into account the structural and temporal kinematics of the hands. First a statistical model is used for skin colour segmentation to roughly locate hands and faces. Then a neural network is used to reconstruct in 3D the hands and faces. For the filtering and the reconstruction we have used the growing neural gas algorithm which can preserve the topology of an object without restarting the learning process. Experiments conducted on our own database but also on four benchmark databases (Stirling’s, Alicante, Essex, and Stegmann’s) and on deaf individuals from normal 2D videos are freely available on the BSL signbank dataset. Results demonstrate the validity of our system to solve problems of face and hand segmentation and reconstruction under different environmental conditions.

## 1 Introduction

Over the last decades, there has been an increasing interest in using neural networks and computer vision techniques to allow users to directly explore and manipulate objects in a natural and intuitive environment without the use of electromagnetic tracking systems. Such a sensor-free human–machine interaction system is simpler and more flexible and therefore more adaptable for a broad range of applications in all aspects of life in a modern society: from gaming and robotics to medical tasks. Considering recent progress in the computer vision field, there has been an increasing interest in the medical domain [55], especially in relation to screening or assessment of acquired neurological impairments associated with motor changes in older individuals, such as dementia, stroke and Parkinson’s disease. Deploying hand gesture recognition or hand trajectory tracking systems is one of the most practical approaches, which is also attributed to their natural and intuitive quality. Moreover, with the recent rise of non-intrusive sensors (e.g. Microsoft Kinect, Leap motion) gesture recognition and face detection have added an extra dimension to human–machine interaction. However, the images captured of hand gestures, which are effectively a 2D projection of a 3D object, can become highly complex for any recognition system. Systems that follow a model-based method [1, 46] require an accurate 3D model that efficiently captures the hand’s articulation in terms of its high degrees of freedom (DOF) and elasticity. The main drawback of a model-based method is that it requires massive calculations, making it unrealistic for real-time implementation. Since this method is too complicated to implement, the most widespread alternative is the feature-based method [26] where features such as the geometric properties of the hand or face are analysed using either neural networks (NNs) [47, 52] or stochastic models such as hidden Markov models (HMMs) [11, 49]. This is feasible because of the emergence of cheap 3D sensors capable of providing a real-time data stream and therefore enabling feature-based computation of three-dimensional environment properties like curvature, an approach closer to human learning procedures.

Another approach for both faces and hands is to use a skin colour classifier [27]. Colour processing is much faster than processing other facial features. Under certain lighting conditions, colour is orientation invariant. It reduces the search space for human targets by segmenting images into skin and non-skin regions based on pixel colour. However, tracking human faces using colour as a feature has several problems: the colour representation of a face obtained by a camera is influenced by many factors (luminance, object movement, etc.); different cameras produce significantly different colour values, even for the same person under the same lighting conditions; and skin colour differs from person to person. Nevertheless, many researchers have worked with skin colour segmentation. Ghazali et al. [17] proposed a skin Gaussian model in YCgCr colour space for detecting human faces. However, the technique still produces false positives in complex backgrounds. Subasic et al. [45] used mean shift and AdaBoost to segment and label human faces in complex background images. However, the image database they used for evaluation testing consists of just a single frontal face detection. Khan et al. [24] noted that detection rate is dependent on skin colour selection, which can be improved by using an appropriate lighting correction algorithm. Zakaria and Suandi [54] reported that skin colour detection failure due to illumination effects increases the false positive rate. Additionally, the processing time also increases because many face candidates are sent to the classifier for verification purposes. Yan et al. [51] proposed a hierarchy based on use of a structure model and structural support vector machine (SVM) in learning to handle global variation in the appearance of human faces. However, a hierarchical structure approach in face detection architecture needs integration between one or more classifications, and this increases the overall processing time.

In order to use colour as a feature for face or hand tracking, we have to solve these problems. In the learning framework, the initialisation of the object is crucial. The main approach is to find a suitable means of segmentation that separates the object of interest from the background. While a great deal of research has been focused on efficient detectors and classifiers, little attention has been paid to efficiently acquiring and labelling suitable training data. One method is to partition the image into regions. Each region of interest is spatially contiguous and the pixels in that region are of the same kind with respect to the predefined criteria. However, the segmentation process itself may be time-consuming as it is usually performed manually [3]. Obtaining a set of training examples automatically is a more difficult task. Existing approaches to minimise labelling effort [28, 33, 39, 40] use a classifier which is trained in a small number of examples. The classifier is then applied by means of a training sequence, and the detected patches are added to the previous set of examples. However, to learn the model for feature position and appearance, a great number (e.g., 10,000 images) of hand-labelled face images are needed. A further disadvantage of these approaches is that either manual initialisation [21] or a pre-trained classifier is needed to initialise the learning process. With a sequence of images, these disadvantages can be avoided by using an incremental model.

In this work, what we are interested in is the accurate initialisation of the first frame of the neural network model. If this is achieved, by performing an accurate segmentation in relation to the background so that the regions that represent the foreground (objects of interest) can be classified by the learning model, then the network preserves the topology in consecutive frames and acceleration to the network is achieved. The key to successful segmentation relies on reducing meaningless image data. We achieve automatic segmentation by taking into consideration that human skin has a relatively unique colour, and we apply appropriate parametric skin distribution modelling. Although the use of the SOM-based techniques of neural gas (NG) [31], growing cell structures (GCS) [13] and growing neural gas (GNG) [14] for various data inputs has already been studied and successful results have been reported [8, 19, 20, 37, 44, 46], some limitations still persist. Most of these studies have assumed noise-free environments and low complexity distributions. Therefore, applying these methods to challenging real-world data obtained using noisy 2D^1^ and 3D^2^ sensors is our main study. These particular noninvasive sensors have been used in the associated experiments and are typical, contemporary technology.

The remainder of this paper is organised as follows. Section 2 presents the initialisation of the object using probabilistic colour models. Section 3 provides a description of the original GNG algorithm and the modifications for topology preservation in 3D. In Sect. 4, a set of experimental results is presented for various datasets before conclusions are drawn in Sect. 5.

## 2 Approach and methodology

The growing neural gas (GNG) [14] is an incremental neural model able to learn the topological relations of a given set of input patterns by means of competitive Hebbian learning [30]. Unlike other methods, the incremental character of this model avoids the necessity of previously specifying the network size. On the contrary, from a minimal network size, a growth process takes place, where new neurons are inserted successively using a particular type of vector quantisation [14]. With this approach, however, the problem of background modelling takes central stage, where the goal is to get a segmentation of the background, i.e. the irrelevant part of the scene and the foreground. If the model is accurate, the regions that represent the foreground (objects of interest) can then be extracted. This problem also plays a central role, since we are interested in setting the initial frame for the GNG algorithm.

### 2.1 Background modelling

We subdivide background modelling methods into two categories: (1) background subtraction methods; and (2) statistical methods. In background subtraction methods, the background is modelled as a single image and the segmentation is estimated by thresholding the background image and the current input image. Background subtraction can be done either using a frame differencing approach or using a pixel-wise average or median filter over *n* number of frames. In statistical methods, a statistical model for each pixel describing the background is estimated. The more the variance of the pixel values, the more accurate the multi-modal estimation. In the evaluation stage of the statistical models, the pixels in the input image are tested if they are consistent with the estimated model. The most well-known statistical models are the eigenbackgrounds [9, 34], the Single Gaussian Model (SGM) [6, 50] and Gaussian Mixture Models (GMM) [12, 42].

The methods based on background subtraction are limited in more complicated scenarios. For example, if the foreground objects have similar colour to the background, these objects cannot be detected by thresholding. Furthermore, these methods only adapt to minor changes in environmental conditions. Changes such as turning on the light cannot be captured by these models. In addition, these methods are limited to segmenting the whole object from the background, although for many tasks, such as face recognition, gesture tracking, etc., specific parts need to be detected. Since most image sources (i.e. cameras) provide colour images, we can use this additional information in our model for the segmentation of the first image. This information can then be stored in the network structure and used to detect changes between consecutive frames.

#### 2.1.1 Probabilistic colour models: single Gaussian

Image segmentation based on colour is studied by many researchers especially in applications of object tracking [5, 7, 29, 41] and human–machine interaction [4, 15]. Also, a great deal of research has been done in the field of skin colour segmentation [22, 23, 35] since the human skin can create clusters in the colour space and thus be described by a multivariate normal distribution. First, we attempt to model skin colour using a Single Gaussian distribution. For the skin domain, we have used the Stirling^3^ and Essex^4^ databases. With SGM, the model can be obtained via the maximum likelihood criterion which looks for the set of parameters (mean and covariance) that maximises the likelihood function. The likelihood function has a single maximum, and the estimates *μ* and *Σ* for the mean vector and the covariance matrix are obtained analytically by

(1) $$\mu =\frac{1}{T}\sum_{t=1}^{T}x_{t}$$

(2) $$\Sigma =\frac{1}{T}\sum_{t=1}^{T}\left(x_{t}-\mu \right){\left(x_{t}-\mu \right)}^{T}$$

where *μ* is the estimated mean vector, *Σ* is the estimated covariance matrix, *T* is the number of observations in the sample set, and *x_t_* is the *t*th observation. The resulting Gaussian PDF that fits the data is given by

(3) $$p\left(x|\mu ,\Sigma \right)=\frac{1}{{\left(2\pi \right)}^{\frac{d}{2}}{\left(\text{det}\left(\Sigma \right)\right)}^{\frac{1}{2}}}\times \text{exp}\left(-\frac{1}{2}D^{2}\right)$$

where

(4) $$D^{2}=\left(x-\mu \right)\Sigma^{-1}{\left(x-\mu \right)}^{T}$$

is the square Mahalanobis distance and *d* is the dimensionality of the Gaussian function (which is 2 in our case).

**Fig. 1:**
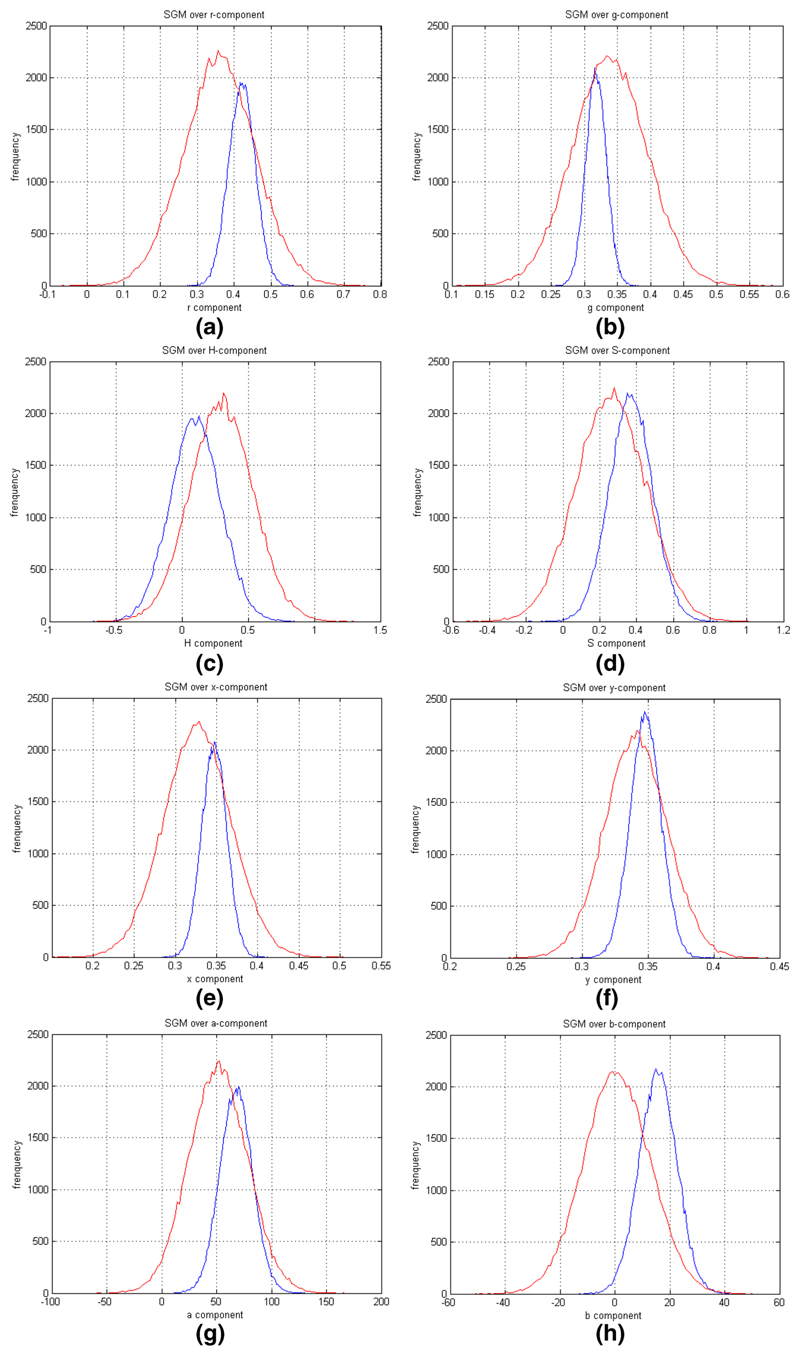
The blue line represents skin and the red line represents background SGM. **a** Estimated SGM for r-component of nRGB. **b** Estimated SGM for g-component of nRGB. **c** Estimated SGM for H-component of HSV. **d** Estimated SGM for S-component of HSV. **e** Estimated SGM for x-component of CIE *X*, *Y*, *Z*. **f** Estimated SGM for y-component of CIE *X*, *Y*, *Z*. **g** Estimated SGM for a-component of CIE L*, a*, b*. **h** Estimated SGM for b-component of CIE L*, a*, b* (colour figure online)

Figure 1 illustrates the SGM model applied to different colour spaces (nRGB, HSV, CIE *X*, *Y*, *Z*, and CIE L*, a*, b*). It is evident that the SGM model covers the entire area of the distribution for both skin and background. In some colour spaces, the differentiation is greater, but the overlapping between skin and non-skin regions is sufficient to produce high false positive rate (FPR). As such, an SGM distribution cannot model all possible variation in the skin colour data. The existing approaches [6, 50] were extended by using Gaussian Mixture Models [36, 53].

#### 2.1.2 Probabilistic colour models: Gaussian mixture model

Below we summarise the steps involved in a MG skin colour model.

- Firstly, the variance caused by the intensity is removed. This is achieved by normalising the data or by transforming the original pixel values into a different colour space (e.g., rg colour space [38] or HSV colour space [35]).
- Secondly, a colour histogram is computed, which is used to estimate an initial mixture model.
- Finally, a Gaussian mixture model is estimated, which can efficiently be done by applying the iterative EM-algorithm [10].

Assume a Gaussian mixture model:

(5) $$\theta =\left\{\pi^{\left(j\right)},\varphi^{\left(j\right)}=\left\{\mu^{\left(j\right)},\Sigma^{\left(j\right)}\right\};j=1,\ldots \;\ldots ,J\right\}$$

where *π*^(*j*)^ denotes the prior probability of expert *j*, and *φ*^(*j*)^ = {*μ*^(*j*)^, *Σ*^(*j*)^} denotes the parameters mean *μ*^(*j*)^, and full-rank covariance matrix *Σ*^(*j*)^ of the expert. The GMM’s output is given by:

(6) $$p\left(x_{t}|\theta \right)=\sum_{j=1}^{J}\pi^{\left(j\right)}p\left(x_{t}|\delta_{t}^{\left(j\right)}=1,\varphi^{\left(j\right)}\right)$$

where

(7) $$\begin{array}{l} p\left(x_{t}|\delta_{t}^{\left(j\right)}=1,\varphi^{\left(j\right)}\right)={\left(2\pi \right)}^{-\frac{D}{2}}{\left|\Sigma^{\left(j\right)}\right|}^{-\frac{1}{2}}\\ \text{exp}\;\left\{-\frac{1}{2}{\left(x_{t}-\mu^{\left(j\right)}\right)}^{T}{\left(\Sigma^{\left(j\right)}\right)}^{-1}\left(x_{t}-\mu^{\left(j\right)}\right)\right\} \end{array}$$

is the *j*th Gaussian density of the GMM. The method for determining the parameters of a Gaussian mixture model from a data set is based on maximising the data likelihood.

(8) $$L\left(X|\theta_{n}\right)\equiv \text{log}\;p\left(X|\theta_{n}\right)=\sum_{Z}P\left(Z|X,\theta_{n}\right)\text{log}\;p\left(X|\theta_{n}\right)$$

Because the likelihood is a differentiable function, it is possible to use general purpose optimisation algorithms such as the EM algorithm for fast convergence. After the initialisation of *θ*_0_, the EM iteration is as follows:

1. **E-step** As we do not know the class labels, but do know their probability distribution, what we can do is to use the expected values of the class labels given the current parameters. For the *n*th iteration, we form the function *Q*(*θ*|*θ_n_*) as follows:
  (9) $$\begin{array}{l} Q\left(\theta |\theta_{n}\right)=E\left\{\text{log}\;p\left(Z,X|\theta \right)|X,\theta_{n}\right\}\\ \;=\sum_{t=1}^{T}\sum_{j=1}^{J}E\left\{\delta_{t}^{\left(j\right)}|x_{t},\theta_{n}\right\}\;\text{log}\;[p\left(x_{t}|\delta_{t}^{\left(j\right)}=1,\varphi^{\left(j\right)}\right)\pi^{\left(j\right)}] \end{array}$$
  and define
  (10) $$h_{n}^{\left(j\right)}\left(x_{t}\right)\equiv E\left\{\delta_{t}^{\left(j\right)}|x_{t},\theta_{n}\right\}=P\left(\delta_{t}^{\left(j\right)}=1|x_{t},\theta_{n}\right)$$
  Using Bayes’ theorem, we can calculate $h_{n}^{\left(j\right)}\left(x_{t}\right)$ as:
  (11) $$h_{n}^{\left(j\right)}\left(x_{t}\right)=\frac{p\left(x_{t}|\delta_{t}^{\left(j\right)}=1,\varphi_{n}^{\left(j\right)}\right)\pi_{n}^{\left(j\right)}}{\sum_{k=1}^{J}p\left(x_{t}|\delta_{t}^{\left(k\right)}=1,\varphi_{n}^{\left(k\right)}\right)\pi_{n}^{\left(k\right)}}$$
  which is actually the expected posterior distribution of the class labels given the observed data. In other words, the probability that *x_t_* belongs to group *j* given the current estimates *θ_n_* is given by $h_{n}^{\left(j\right)}\left(x_{t}\right).$ The calculation of *Q* is the E-step of the algorithm and determines the best guess of the membership function $h_{n}^{\left(j\right)}\left(x_{t}\right).$
2. To compute the new set of parameter values of *θ* (denoted as θ*), we optimise *Q*(*θ*|*θ_n_*); such as θ* = arg max_*θ*_ *Q*(*θ*|*θ_n_*). This is the **M-step** of the algorithm.
  Specifically, the steps are:
  - Maximise *Q*(*θ*|*θ_n_*) with respect to *θ* to find θ*.
  - Replace *θ_n_* by θ*.
  - Increment *n* by 1 and repeat the E-step until convergence.

To determine *μ*^(*k*)*^, differentiate *Q* with respect to *μ*^(*k*)^ and equate to zero $\left(\frac{\vartheta Q\left(\theta |\theta_{n}\right)}{\vartheta \mu^{\left(k\right)}}=0\right)$ which gives:

(12) $$\mu^{{\left(k\right)}^{*}}=\frac{\sum_{t=1}^{T}h_{n}^{k}\left(x_{t}\right)x_{t}}{\sum_{t=1}^{T}h_{n}^{k}\left(x_{t}\right)}$$

To determine *Σ*^(*k*)*^, differentiate *Q* with respect to *Σ*^(*k*)^ and equate to zero $\left(\frac{\vartheta Q\left(\theta |\theta_{n}\right)}{\vartheta \Sigma^{\left(k\right)}}=0\right)$ which gives:

(13) $$\Sigma^{{\left(k\right)}^{*}}=\frac{\sum_{t=1}^{T}h_{n}^{k}\left(x_{t}\right)\left(x_{t}-\mu^{\left(k\right)*}\right){\left(x_{t}-\mu^{\left(k\right)*}\right)}^{T}}{\sum_{t=1}^{T}h_{n}^{k}\left(x_{t}\right)}$$

To determine *π*^(*k*)*^, maximise *Q*(*θ*|*θ_n_*) with respect to *π*^(*k*)^ subject to the constraint $\Sigma_{j=1}^{J}\pi^{j}=1$ which gives:

(14) $$\pi^{{\left(k\right)}^{*}}=\frac{1}{T}\sum_{t=1}^{T}h_{n}^{k}\left(x_{t}\right)$$

**Fig. 2:**
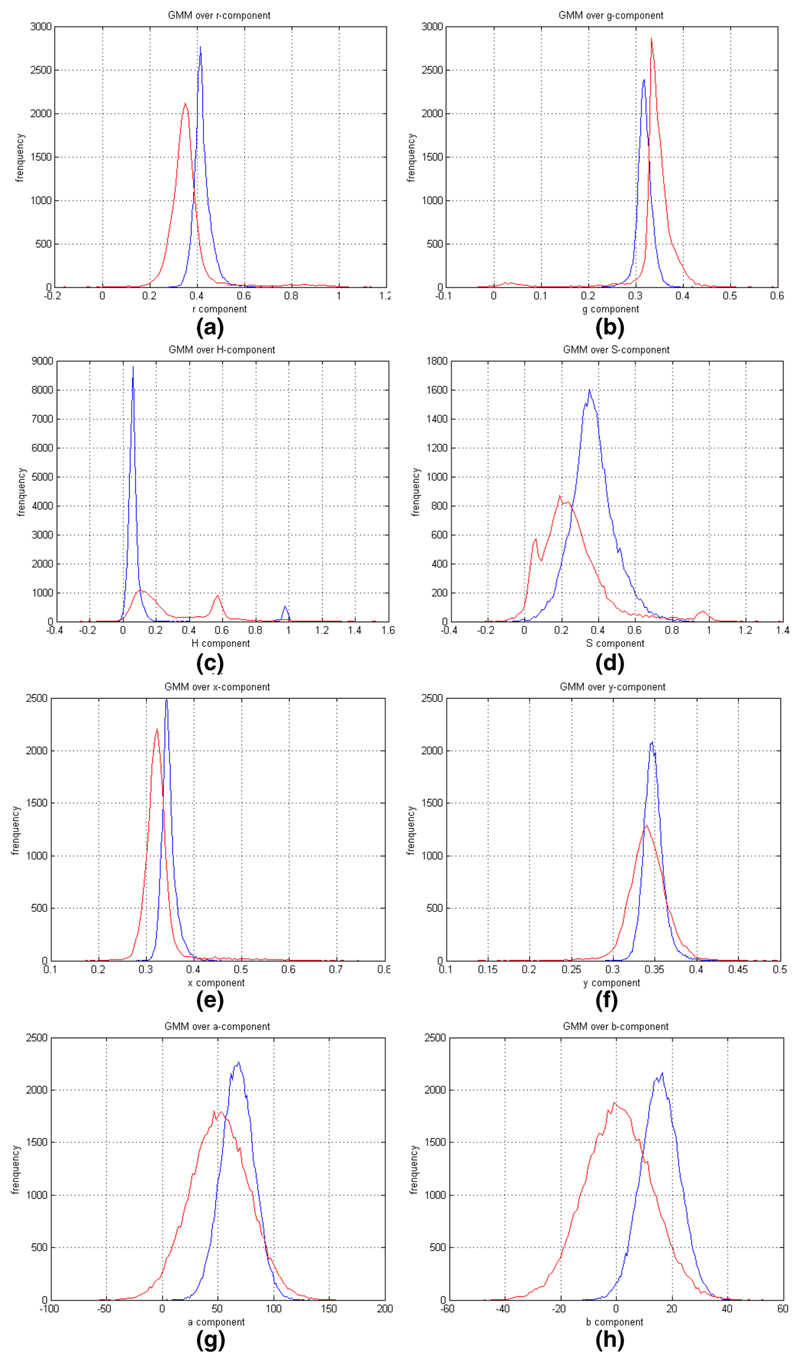
The blue line represents skin and the red line represents background GMM. **a** Estimated GMM for r-component of nRGB. **b** Estimated GMM for g-component of nRGB. **c** Estimated GMM for H-component of HSV. **d** Estimated GMM for S-component of HSV. **e** Estimated GMM for x-component of CIE *X*, *Y*, *Z*. **f** Estimated GMM for y-component of CIE *X*, *Y*, *Z*. **g** Estimated GMM for a-component of CIE L*, a*, b*. **h** Estimated GMM for b-component of CIE L*, a*, b* (colour figure online)

The two steps are repeated until the likelihood does not change significantly or the maximum number of iterations is reached. The GMMs obtained after 5 EM iterations for the different colour spaces (nRGB, HSV, CIE *X*, *Y*, *Z*, and CIE L*, a*, b*) are shown in Fig. 2.

The results were obtained from a database containing approximately one half million pixels.

**Fig. 3:**
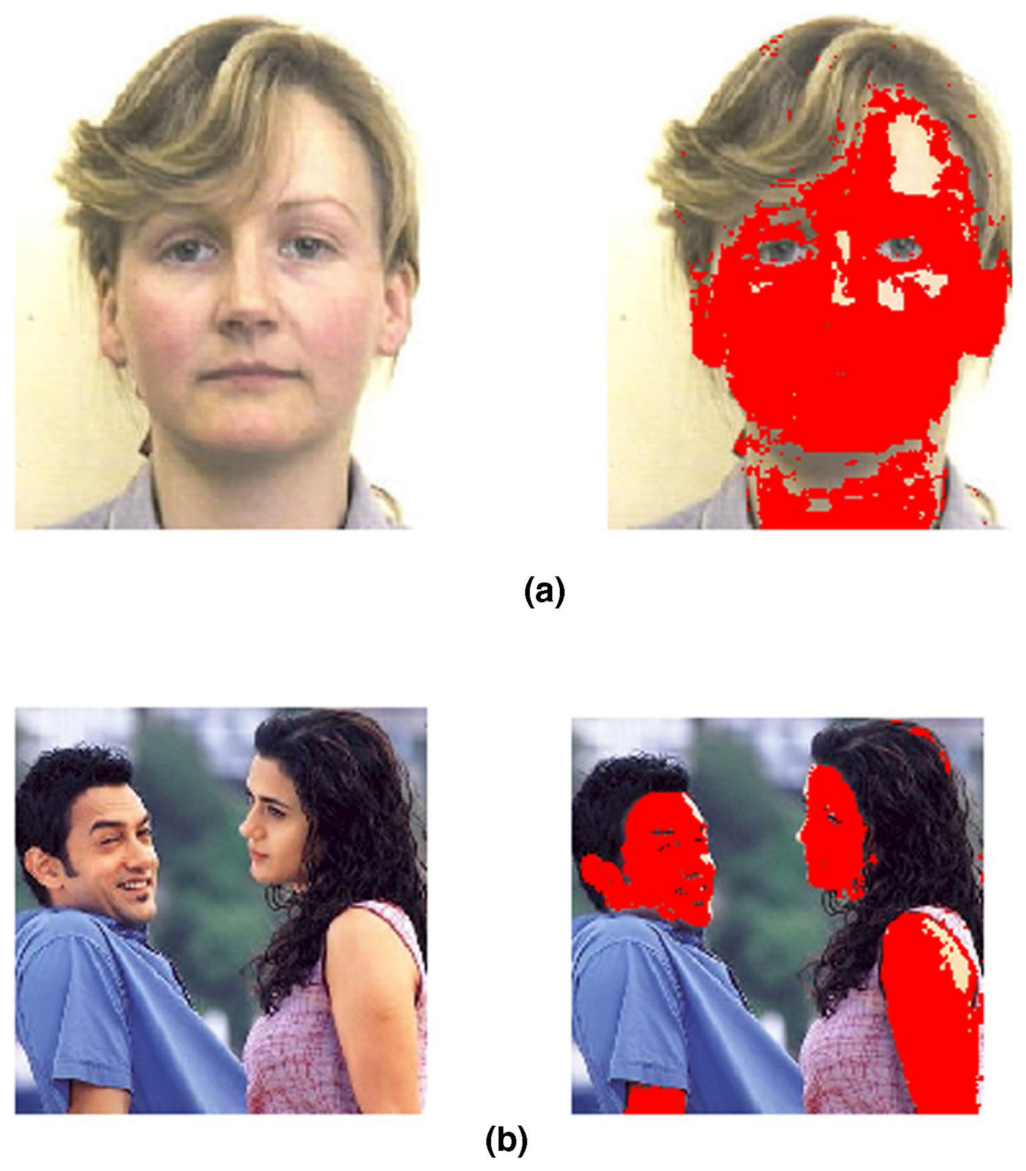
**a**, **b** Image segmentation on simple background based on skin colour information. Skin area marked in red (colour figure online)

**Fig. 4:**
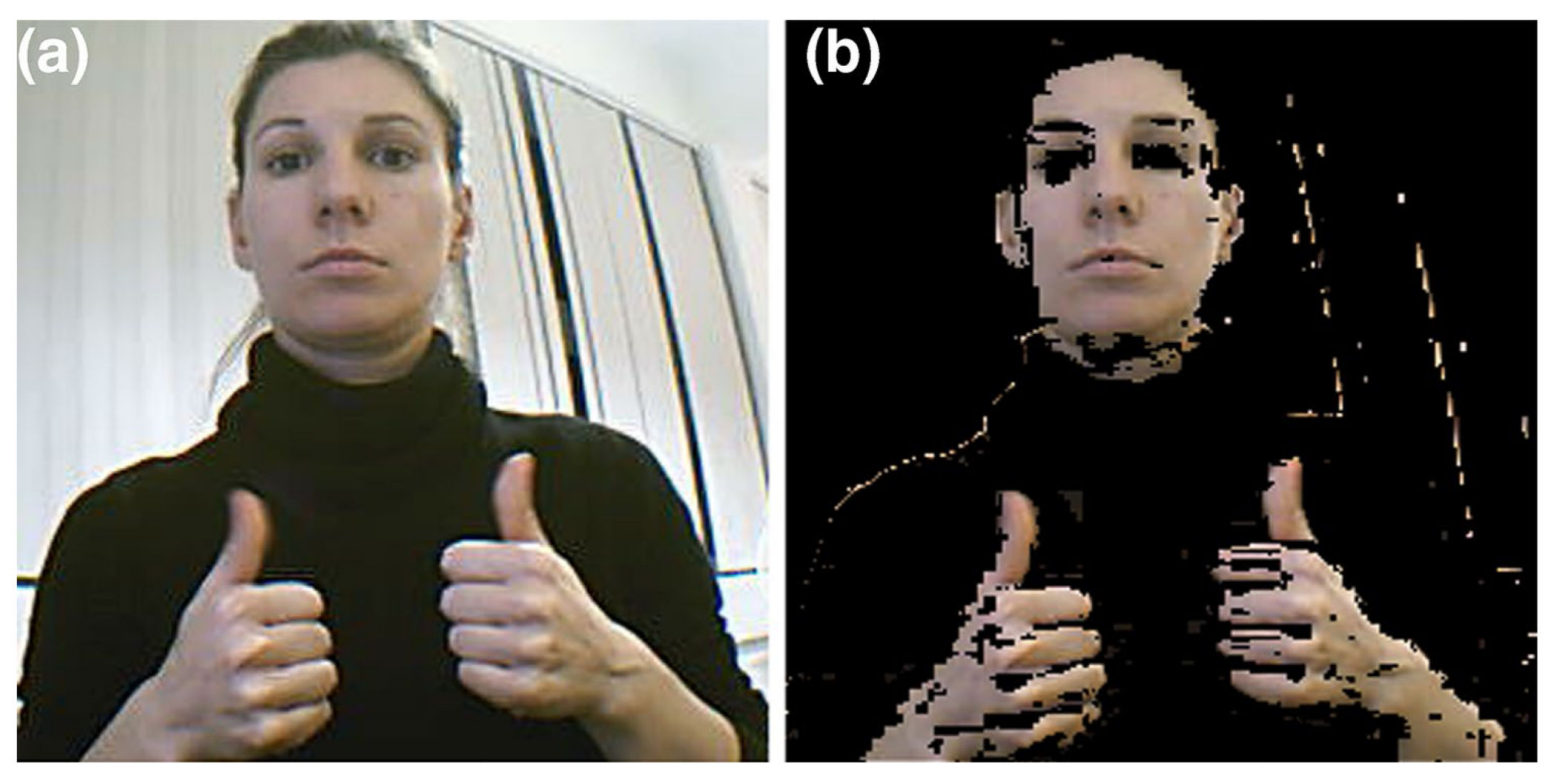
Image segmentation on a different background based on skin colour information. **a** original input image, and **b** probability map for the skin colour

Figures 3 and 4 show the GMM probability map for CIE L*, a*, b* skin colour against different backgrounds, which can then be used to initialise the network for the GNG algorithm.

**Fig. 5:**
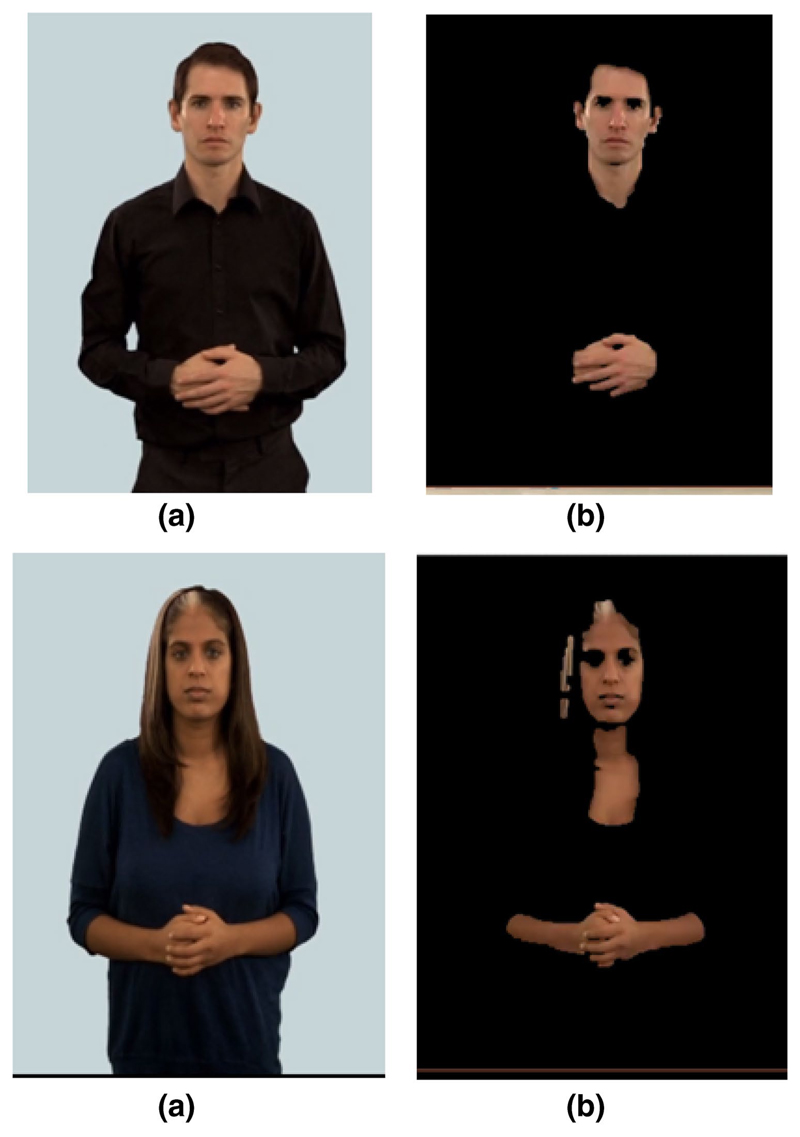
Image segmentation by skin colour in the BSL Signbank dataset. **a** Original input image, and **b** probability map for the skin colour

Figure 5 shows another example of the probability map for skin colour in deaf individuals, using normal 2D videos freely available from the BSL SignBank dataset,^5^ which can then be implemented with the specific goal of developing an automated dementia screening toolkit. In all three examples, the colour space used to represent the input image plays an important part in the segmentation. As we have seen above, some models are more perceptually uniform than others and some separate out information such as luminance and chrominance.

**Fig. 6:**
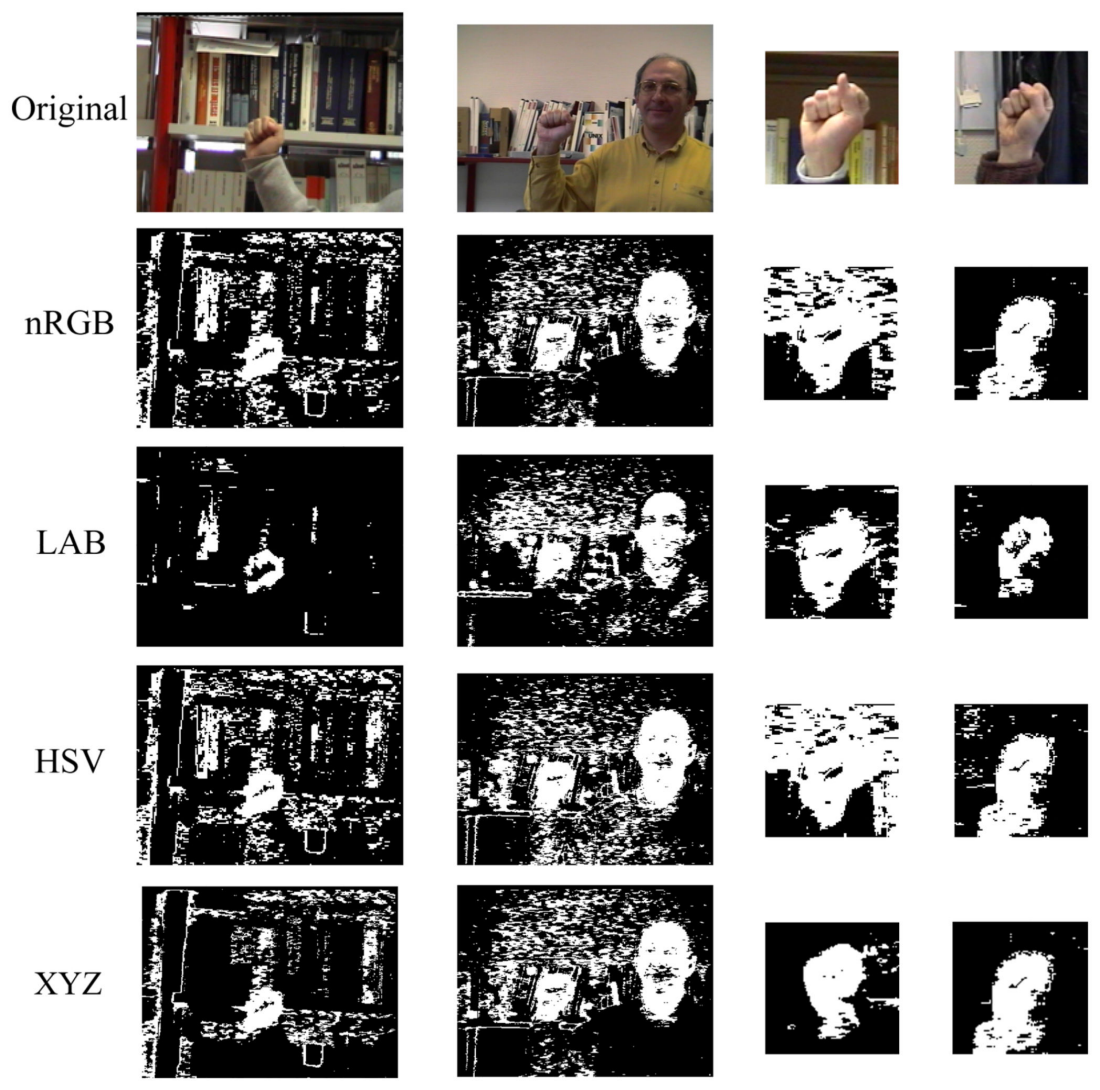
Skin colour images in cluttered backgrounds. Top row shows the original images followed by the various colour spaces

At each node of the network, we experimented with more cluttered backgrounds with the perceptually uniform colour model CIE L*, a*, b*, and the non-perceptually uniform colour models, nRGB, and CIE *X*, *Y*, *Z*. We also experimented with the HSV colour model, which separates the brightness component from hue and saturation, to compensate for changes in illumination (Fig. 6). Unlike CIE L*, a*, b*, HSV is not perceptually mapped to the human visual system, meaning changes in colour values are not proportional to changes in the perceived significance of the change. A detailed discussion of the different colour models can be found in [23, 43, 48].

**Fig. 7:**
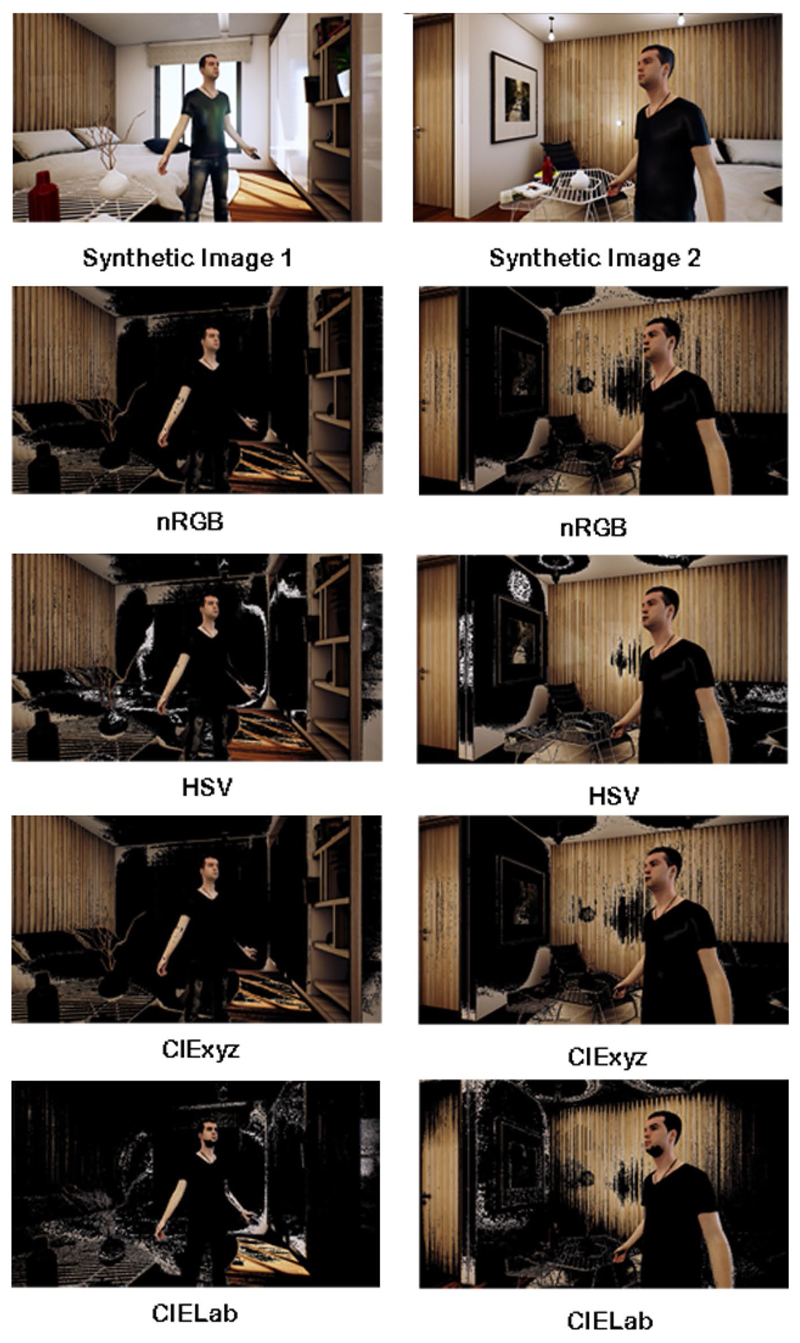
Image segmentation on two synthetic images generated with UnrealROX [32]. From top to bottom, original input image, and probability maps for the skin colour after applying the four different chrominance models

In order to address some of the challenges we face in real environments with changes in illumination, shadows and more cluttered backgrounds, we have tested the different chrominance models on two synthetic images we have generated with our virtual environment UnrealROX [32]. Figure 7 shows the segmentation of the skin colour after applying the four different chrominance models and getting the skin probability with a GMM. Qualitative results demonstrate CIE L*, a*, b*, as the most efficient for skin segmentation since it is the most perceptually mapped to the visual system.

Given consideration of perceptual uniformity our best option is the CIE L*, a*, b* colour space. Thus, we converted the RGB values to the CIE L*, a*, b* values. The colour conversion from RGB to CIE L*, a*, b* is obtained by undergoing a linear conversion from RGB to CIE *X*, *Y*, *Z*, and a nonlinear conversion from CIE *X*, *Y*, *Z* to CIE L*, a*, b*.

(15) $$\left[\begin{array}{l} X\\ Y\\ Z \end{array}\right]=\left[\begin{array}{lll} 0.433910 & 0.376220 & 0.189860\\ 0.212649 & 0.715169 & 0.072182\\ 0.017756 & 0.109478 & 0.872915 \end{array}\right]*\left[\begin{array}{l} R\\ G\\ B \end{array}\right]$$

(16) $$\begin{array}{l} L^{*}=116*f\left[\frac{Y}{Y_{n}}\right]-16\\ \;a^{*}=500*\left[f\left[\frac{X}{X_{n}}\right]-f\left[\frac{Y}{Y_{n}}\right]\right]\\ \;b^{*}=200*\left[f\left[\frac{Y}{Y_{n}}\right]-f\left[\frac{Z}{Z_{n}}\right]\right] \end{array}$$

where

(17) $$f\left(r\right)=\left\{\begin{array}{ll} r^{\frac{1}{3}} & \text{if}\;r>0.008856\\ 7.7867*r+\frac{16}{116} & \text{if}\;r\leq 0.008856 \end{array}\right\}$$

The *X_n_*, *Y_n_* and *Z_n_* refer to the CIE *X*, *Y*, *Z* values for a specified white point. To get the learning process started, we need a simple but robust method to obtain the topology preserving graph (TPG) with as little user intervention as possible. Henceforth, the topology preserving graph TPG = 〈*N*, *C*〉 is defined with a vertex (neurons) set *N* and an edge set *C* that connects them. The self-organising neural network growing neural gas (GNG) is used to filter out non-face candidates and detect and reconstruct the face and or the hands.

## 3 Growing neural gas (GNG) algorithm in 2D and 3D

In order to determine where to insert new neurons, local error measures are gathered during the adaptation process and each new unit is inserted near the neuron which has the highest accumulated error. At each adaptation step, a connection between the winner and the second-nearest neuron is created as dictated by the competitive Hebbian learning algorithm. This is continued until an ending condition is fulfilled, as for example evaluation of the optimal network topology based on the topographic product [16]. This measure is used to detect deviations between the dimensionalities of the network and that of the input space, detecting folds in the network and indications that it is trying to approximate to an input manifold with different dimensions. In addition, in a GNG network the learning parameters are constant in time, in contrast to other methods where learning is based on decaying parameters.

The network is specified as:

- A set *N* of nodes (neurons). Each neuron *c* ∈ *N* has its associated reference vector *w_c_* ∈ *R^d^*. The reference vectors can be regarded as positions in the input space of their corresponding neurons.
- A set of edges (connections) between pairs of neurons. These connections are not weighted and their purpose is to define the topological structure. The edges are determined using the competitive Hebbian learning algorithm. An *edge ageing scheme* is used to remove connections that are invalid because of the activation of the neuron during the adaptation process.

**Fig. 8:**
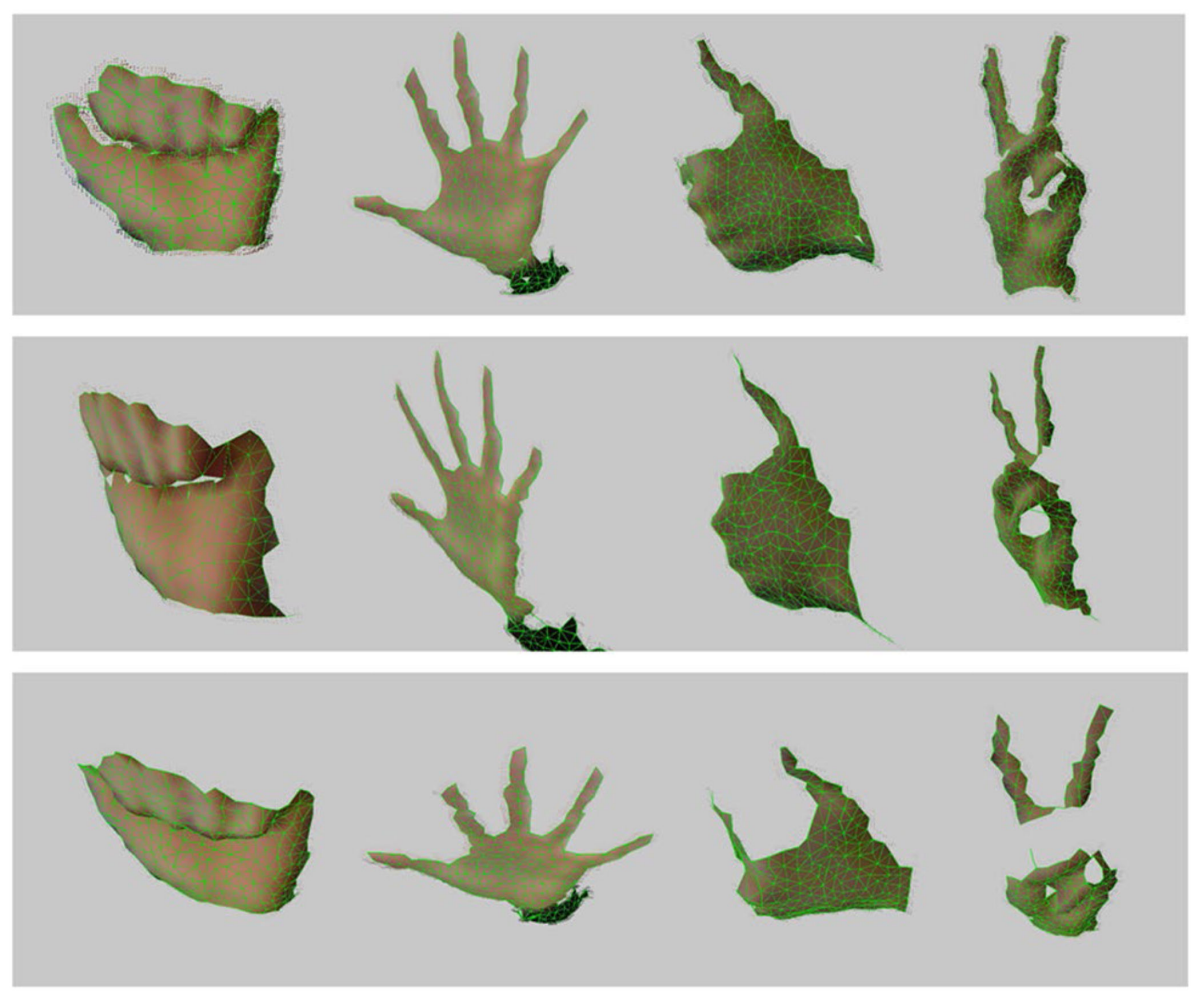
GNG 3D surface reconstructions. 3D reconstruction of different hand poses obtained using the Kinect sensor

The GNG learning is presented in Algorithm 1. With the new advances of low-cost 3D sensors, it is possible to generate more natural gesture-based 3D interactions. However, these systems need to address the problem of 3D tracking of hand joints. In [2], the original GNG algorithm is extended to perform 3D surface reconstruction by considering surface normal information during the learning process. It also modifies the original competitive Hebbian learning process by producing wire-frame 3D representations. In order to obtain 3D information in the form of point clouds, we had to project disparity information obtained from the device to the three-dimensional space using the known geometry of the sensor. The relationship between a disparity map provided by the Kinect sensor and a normalised disparity map is given by *d* = 1/8 · (*d*_off_ − *kd*), where *d* is the normalised disparity, *kd* is the disparity provided by the Kinect and *d*_off_ is a particular offset of a Kinect sensor. Calibration values can be obtained in the calibration step [25]. Figure 8 shows the 3D mesh created using the method discussed in [2].

Algorithm 1 **GNG Algorithm.**

**input :** input vector *x_c_*

**output:** TPG

1. Initialise two vector prototypes *N* = {*c*_1_, *c*_2_} at random positions {*x*_*c*_1__, *x*_*c*_2__}, and the connection set *C*, *C* ⊂ *NxN* to an empty set *C* = 0
2. **while** the current number of prototypes ≤ to the maximum number of prototypes **do**
  (a) **for** every input signal *ξ_w_* **do**
    – Determine the winner prototype *x_v_* and the second nearest *x_υ_* (*x_v_*, *x_υ_* ∈ *N*)
    – Add the squared distance between the input vector *ξ_w_* and the winner *x_v_* to a local accumulated error variable
    – Adjust *x_v_* position and its topological neighbours
    – Update connections between prototypes
    – Remove any dead nodes
      - **if** the current number of prototypes is an integer multiple of a parameter *λ* **then**
        – Add a new prototype
        – Update the connections between the prototypes
        – Decrease local errors
      - **end if**
  (b) **end for**
3. Decrease the error for all prototypes
4. **end while**

It can be seen how this extended algorithm is able to create a coloured 3D mesh that represents the input data. Since point clouds obtained using the Kinect are partial 3D views, the mesh obtained is not complete and therefore the model generated by the GNG is an open coloured mesh.

**Fig. 9:**
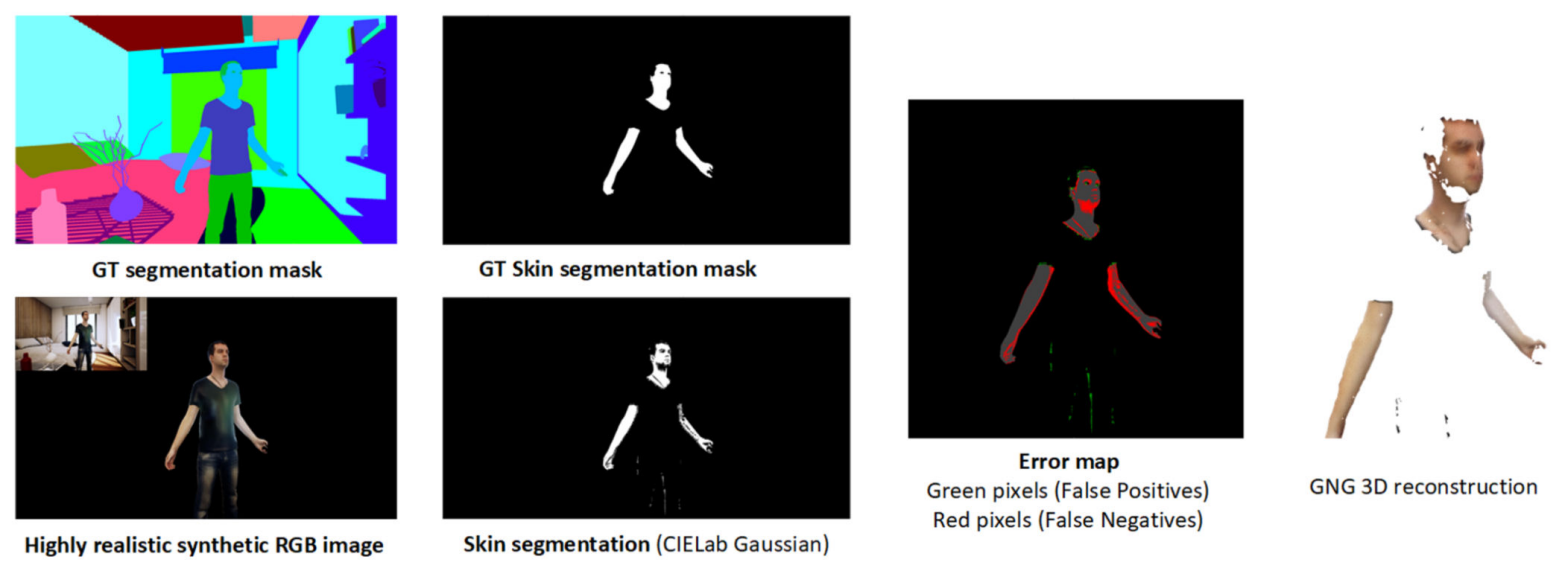
Overview of the whole process using the first synthetic sample. Top row shows the ground truth segmentation and skin masks. Bottom part shows skin segmentation results using CIE L*, a*, b* (Gaussian). The images on the right show the error map of the computed skin mask and the 3D reconstructed mesh using the GNG algorithm

**Fig. 10:**
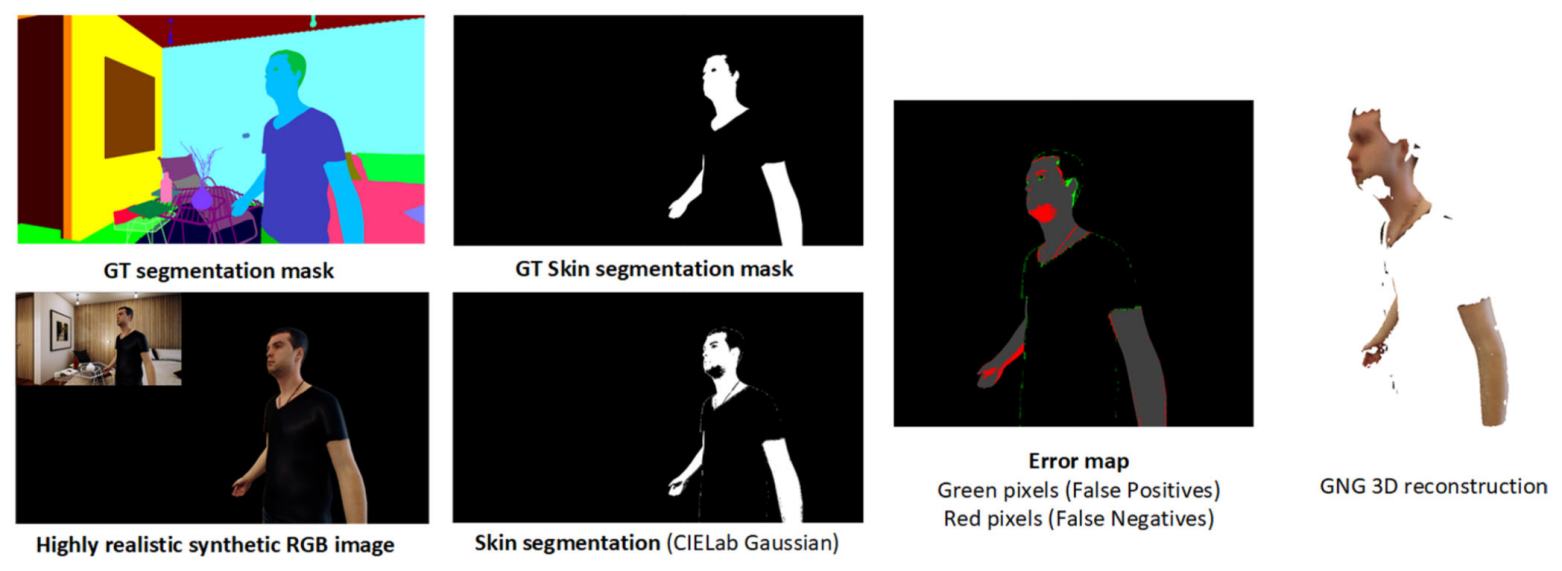
Overview of the whole process using the second synthetic sample. Top row shows the ground truth segmentation and skin masks. Bottom part shows skin segmentation results using CIE L*, a*, b* (Gaussian). The images on the right show the error map of the computed skin mask and the 3D reconstructed mesh using the GNG algorithm

Figures 9 and 10 show the whole process for different input samples that were synthetically generated using UnrealROX [32]. From left to right, we can see the original RGB image and its corresponding ground truth segmentation mask. The ground truth segmentation mask for the skin class and the obtained results using the proposed method (CIE L*, a*, b* Gaussian). Finally, on the right we can see the 3D reconstruction results using as an input the skin mask, RGB and depth images. Using the GNG algorithm we are able to create a simplified coloured mesh from the input data. Since the skin mask has some false positives, the reconstructed mesh also shows small outliers.

## 4 Experiments

In this section, different experiments are shown validating the capabilities of our method (e.g. which statistical model is best for GNG reconstruction) which has also been used in 3D datasets. We tested our system on our own data set (University of Alicante, and University of Westminster) of faces and hands recorded from 15 participants. To create this data set we have recorded images over several days using a simple webcam with image resolution 800 × 600. In total, we have recorded over 112,500 frames, and for computational efficiency, we have resized the images to 300 × 225, 200 × 160, 198 × 234, and 124 × 123 pixels. In addition, experiments were conducted based on two publicly available databases, Mikkel B. Stegmann^6^ and Stirling’s.^7^ All methods have been developed and tested on a desktop machine of 2.26 GHz Pentium IV processor. These methods have been implemented in MATLAB and C++.

**Fig. 11:**
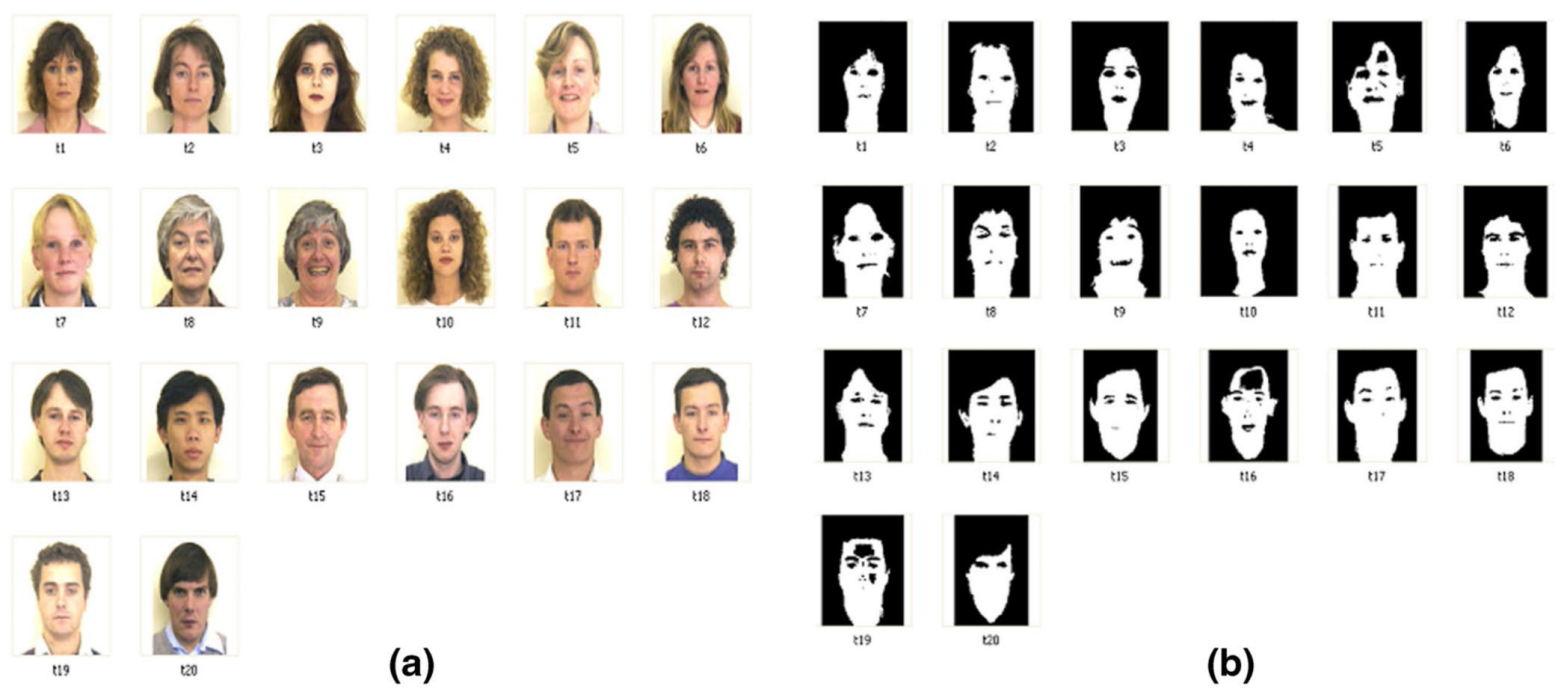
**a** the test image and **b** the ground truth model

Figure 11 shows the ground truth model which we use to measure the error produced by our method. To measure the class probability of skin to non-skin regions we use the positive (TPR) and false positive (FPR) rates as obtained by:

(18) $$\text{TPR}=\frac{\text{TP}}{\text{S}}$$

(19) $$\text{FPR}=\frac{\text{FP}}{\text{NS}}$$

where TP is number of true positive (pixels correctly assigned to the skin class). FP is the number of false positives (non-skin pixels wrongly assigned to skin). *S* is the total of skin pixels and NS the totals of non-skin pixels. These rates TPR and FPR are calculated using both models (SGM and GMM) for all test images. Through this computation we get vector of measures *P* = (TPR, FPR) that express performance of the given model. In order to measure the similarity between the predicted skin colour region and the ground truth region in the two synthetic images, we applied the Intersection over Union (IoU) metric, as a standard performance measure for all the four chrominance models. The IoU metric measures the number of pixels common to both the target and prediction regions, divided by the total number of pixels present across both regions.

(20) $$\text{IoU}=\frac{\text{target}\cap \text{prediction}}{\text{target}\cup \text{prediction}}$$

We also calculated the loss function of our image segmentation tasks in the two synthetic images based on the Dice coefficient calculated as:

(21) $$\text{Dice}=\frac{2|\;\text{target}\cap \text{prediction}\;|}{\left|\;\text{target}\;\right|+\left|\;\text{prediction}\;\right|}$$

Tables 1 and 2 show the results.

For further comparison between the SGM and the GMM model, we used ROC curves for all the test images. For drawing ROC curves, we calculated TPR and FPR for all images using different threshold (threshold value is set as per *P*(*S*|*x*)′). Thus by using *K* different thresholds, we can get *K* point vectors, which, when plotted, results in a ROC curve for the specific model with respect to test image.

**Fig. 12:**
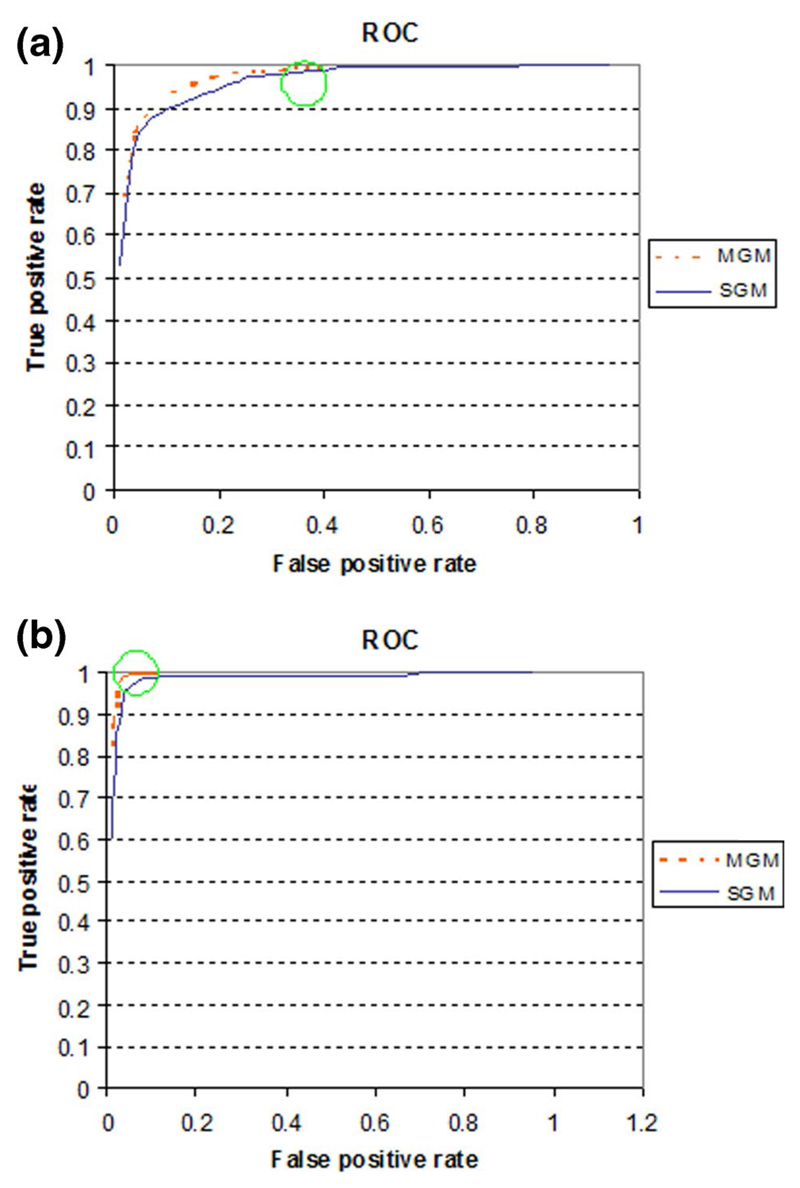
**a** ROC curve for test image 4. **b** ROC curve for test image 17

**Fig. 13:**
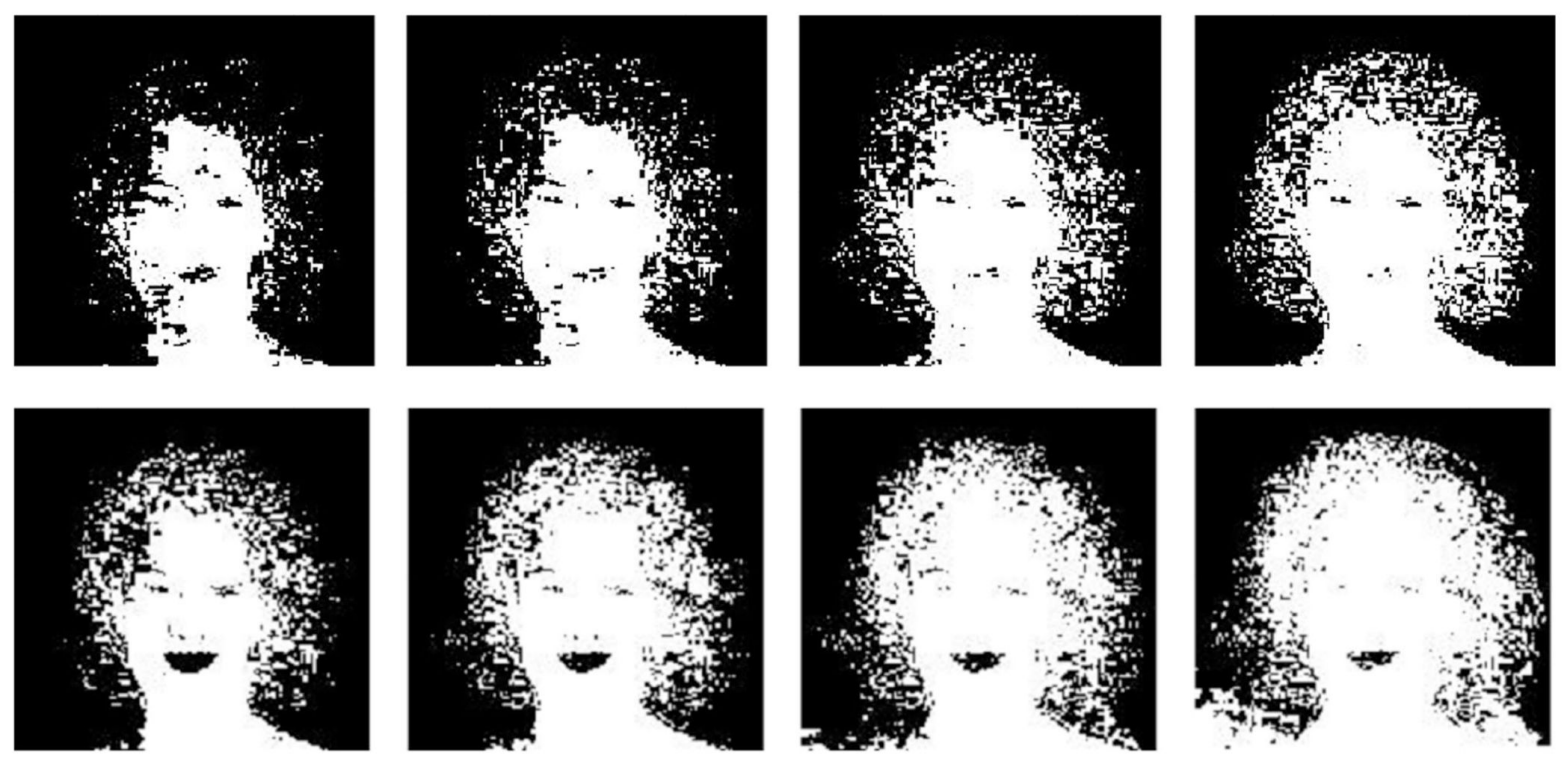
Image 4 threshold representation for GMM (1 row) and SGM (2 row). Threshold values for each column are 0.7, 0.6, 0.5, and 0.4 respectively

**Fig. 14:**
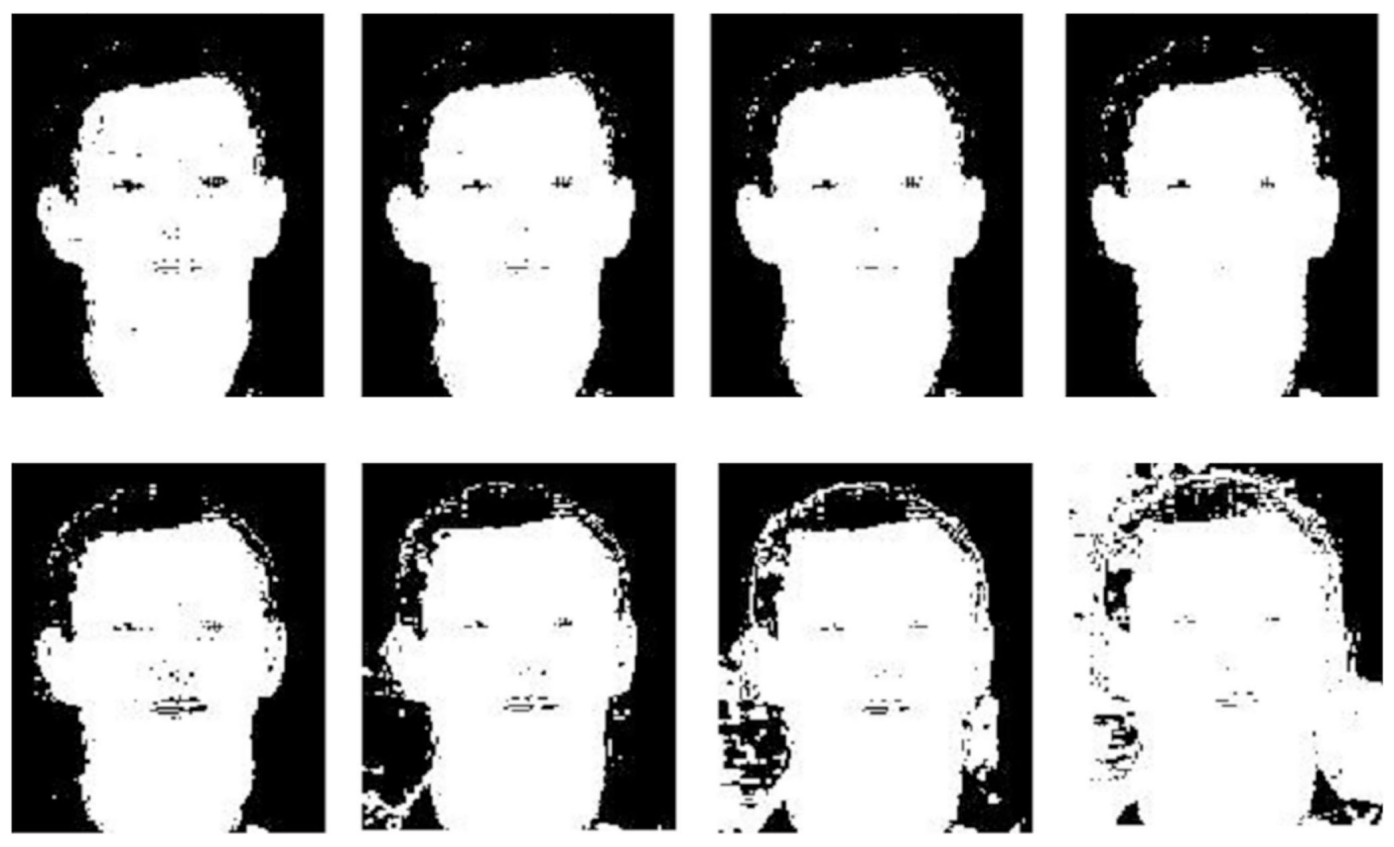
Image 17 threshold representation for GMM (1 row) and SGM (2 row). Threshold values for each column are 0.7, 0.6, 0.5, and 0.4 respectively

Figure 12, shows ROC curves for the test images 4 and 17, respectively, from Fig. 11. It is clear from the ROC curve that both SGM and GMM have almost the same TPR. The FPR in GMM curve (orange dotted line) becomes constant at a certain threshold, as shown by the green circle. However, FPR for SGM (blue line) keeps increasing as the threshold value decreases. FPR reaches nearly to 0.9. Several of the thresholds for images 4 and 17 are shown in Figs. 13 and 14 respectively. From the threshold images, it is very clear that SGM images have a higher FPR rate. Based on the ROC curves for all test images, it is evident that SGM have a very high FPR and thus they perform badly as compared to GMM. Additionally, for several images TPR at a given threshold for SGM was marginally low as compared to GMM. This marginally low TPR will not have any effect on the images with large skin area. Nevertheless for the images with small skin area (small faces), this low TPR can have an adverse effect.

Tables 3 and 4 show calculated TPR and FPR for all four colour spaces using the SGM and GMM statistical models, respectively. For the calculations, we have used a fixed threshold value of 0.55, since it is the middle range value where we can find the highest TPR and lowest FPR for comparison purposes. It can be seen how the CIE L*, a*, b* model outperforms all other three colour spaces since it has the lowest FPR rate among the 20 images, followed by the CIE *X*, *Y*, *Z*. The comparisons demonstrate that GMM outperforms SGM with low FPR rate, which makes it a suitable model for image segmentation especially in cases where hands and faces are involved. This is then used in the initialisation of the first frame of the GNG algorithm.

**Fig. 15:**
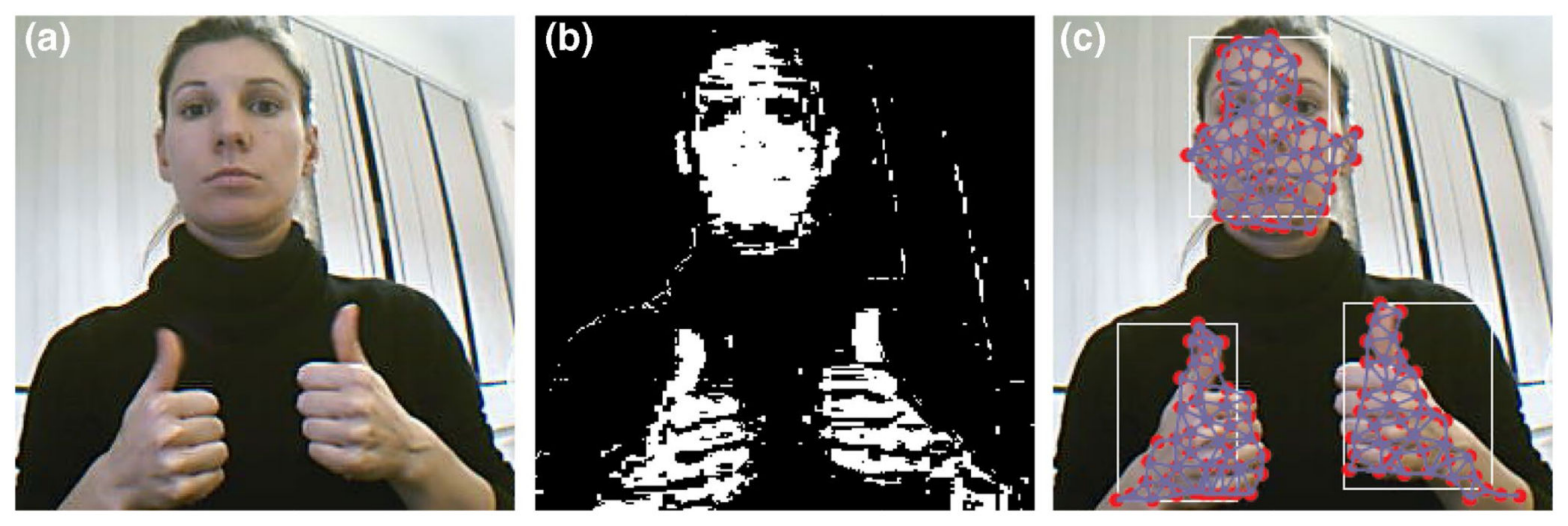
**a** Original image, **b** after applying EM to segment skin region, and **c** hand and face 2D topology with the GNG network

**Fig. 16:**
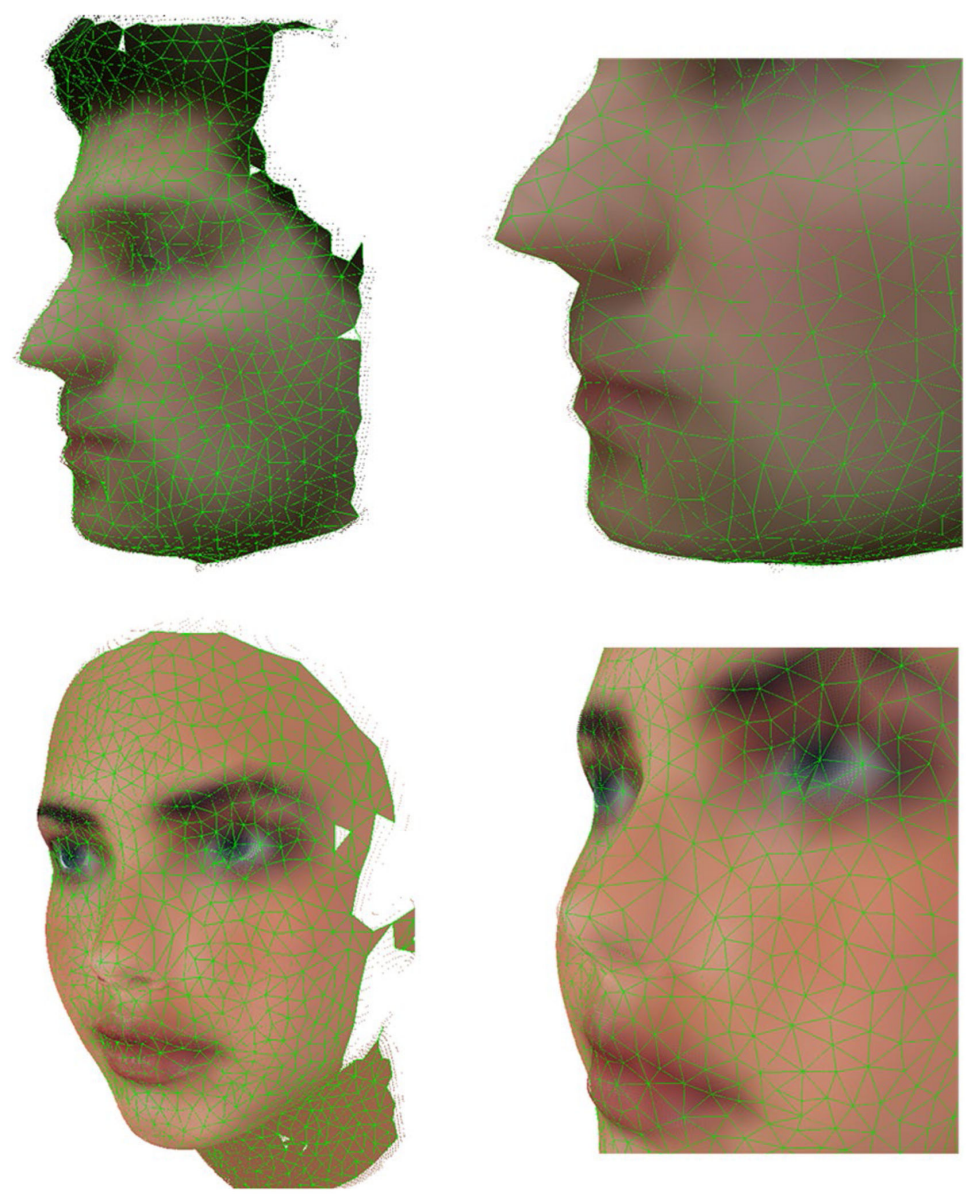
GNG 3D reconstructions. Top: 3D face reconstruction from data obtained using the Kinect sensor. Bottom: 3D face reconstruction from data synthetically generated using Blensor software

Figure 15 shows the correctly detected hands and face after applying EM to segment the skin region. The reconstruction of the 2D topology of both hands and face is done with the GNG algorithm. Figure 16 shows the 3D reconstruction of a human face acquired using the Kinect sensor (top) and the 3D reconstruction of a synthetically generated human face (bottom). Both faces were reconstructed using the GNG for 3D surface reconstruction discussed in Sect. 4. Synthetic data was generated using Blensor software [18], for simulating a virtual Kinect sensor (noise-free).

While 3D downsampling and reconstruction methods like Poisson or Voxelgrid are not able to deal with noisy data, the GNG method is able to avoid outliers and obtain an accurate representation in the presence of noise. This ability is due to the Hebbian learning rule used and its random nature that updates vertex locations based on the average influence of a large number of input patterns.

## 5 Conclusions and future work

In this paper, we have compared the performance of different probabilistic colour models and colour spaces for skin segmentation as an initialisation stage for the GNG algorithm. Based on the capabilities of GNG to readjust to new input patterns without restarting the learning process, we are interested in reducing meaningless image data by taking into consideration that human skin has a relatively unique colour and applying appropriate parametric skin distribution modelling. We concluded that GMM was superior to SGM with lower FPR rates. We also showed that CIE L*, a*, b* colour space outperforms all three other colour spaces since it has the lowest FPR rate among the dataset. **Preprocessing was also used as an initialisation stage in the 3D reconstruction of faces and hands based on the work conducted in [2]. Further work will aim at improving system performance by accelerating GPUs which can then be used for robotic system recognition. Nonetheless, we are currently working, after obtaining a clean segmentation, on hand sign trajectories in order to analyse the sign space envelope (sign trajectories/depth/speed) and facial expressions of deaf individuals. An automated screening toolkit will be beneficial not only to screening of deaf individuals for dementia, but also for assessment of other acquired neurological impairments associated with motor changes, for example, stroke and Parkinson’s disease.

## Figures and Tables

**Table 1:** IoU and Dice score for synthetic image 1

Scene	Frame	Algorithm	Dice score	IoU score
1	1	CIELab*_s_ingle_g_aussian*	**0.874067744**	**0.776282803**
1	1	CIELab*_m_ulti_g_aussian*	0.860429045	0.755023416
1	1	CIExyz*_s_ingle_g_aussian*	0.729085392	0.573655639
1	1	CIExyz*_m_ulti_g_aussian*	0.763614975	0.617600418
1	1	HSV*_s_ingle_g_aussian*	0.74712024	0.596304817
1	1	HSV*_m_ulti_g_aussian*	0.760118655	0.613039857
1	1	nRGB*_s_ingle_g_aussian*	0.773454371	0.630579375
1	1	nRGB*_m_ulti_g_aussian*	0.766449414	0.621317352

Bold indicates the best achieved score

**Table 2:** IoU and Dice score for synthetic image 2

Scene	Frame	Algorithm	Dice score	IoU score
1	2	CIELab*_s_ingle_g_aussian*	0.898387257	0.815509983
1	2	CIELab*_m_ulti_g_aussian*	**0.935972544**	**0.879640743**
1	2	CIExyz*_s_ingle_g_aussian*	0.74361557	0.591862691
1	2	CIExyz*_m_ulti_g_aussian*	0.910774728	0.836158347
1	2	HSV*_s_ingle_g_aussian*	0.930137277	0.86938926
1	2	HSV*_m_ulti_g_aussian*	0.882018032	0.788929152
1	2	nRGB*_s_ingle_g_aussian*	0.901852199	0.821238972
1	2	nRGB*_m_ulti_g_aussian*	0.891526587	0.804274396

Bold indicates the best achieved score

**Table 3:** TPR and FPR rates for all four colour spaces using SGM

HSV		CIE *X, Y, Z*		nRGB		CIE L*, a*, b*	
TPR	FPR	TPR	FPR	TPR	FPR	TPR	FPR
0.5689	0.2395	0.8442	0.1341	0.9156	0.207	0.8902	**0.1031**
0.8947	0.0995	0.9371	0.0596	0.9641	0.1183	0.9471	**0.0485**
0.7538	0.2632	0.8655	0.1985	0.9708	0.2683	0.8015	**0.1583**
0.8568	0.2706	0.8915	0.0969	0.9198	0.1581	0.9015	**0.0889**
0.6378	0.161	0.9019	**0.0709**	0.9492	0.1263	0.9159	0.0719
0.9337	0.2527	0.8587	0.1117	0.9217	0.1669	0.9587	**0.1011**
0.6664	0.1598	0.8383	0.0628	0.8966	0.0813	0.8983	**0.0528**
0.8742	0.0529	0.9247	0.0822	0.9544	0.1352	0.9201	**0.0501**
0.9083	0.109	0.8353	**0.0341**	0.8943	0.0548	0.8853	0.0508
0.512	0.297	0.7843	0.1163	0.8836	0.1787	0.8513	**0.0963**
0.7496	0.0788	0.9172	0.0607	0.9527	0.1021	0.9561	**0.0531**
0.7915	0.0395	0.969	0.0506	0.9834	0.0757	0.9598	**0.0306**
0.8437	0.0789	0.8005	**0.0535**	0.8713	0.0806	0.8995	0.0555
0.65	0.0373	0.7279	0.0401	0.8249	0.0594	0.8271	**0.0324**
0.8503	0.0931	0.9284	0.0864	0.9606	0.1403	0.9684	**0.0804**
0.2353	0.1242	0.7993	0.0483	0.9435	0.0254	0.7803	**0.0382**
0.9598	0.0362	0.9364	0.0349	0.9641	0.054	0.9164	**0.0312**
0.8857	0.0255	0.9501	0.0383	0.9753	0.0556	0.9511	**0.0213**
0.5688	0.0254	0.8118	0.0471	0.9114	0.0984	0.9108	**0.0206**
0.7346	0.0315	0.8503	0.0214	0.9173	0.0409	0.9403	**0.0184**

Bold indicates the best achieved score

**Table 4:** TPR and FPR rates for all four colour spaces using GMM

HSV		CIE *X*, *Y*, *Z*		nRGB		CIE L*, a*, b*	
TPR	FPR	TPR	FPR	TPR	FPR	TPR	FPR
0.9228	0.2374	0.8518	0.2256	0.7931	0.2654	0.8831	**0.2211**
0.9516	0.0654	0.9699	0.0837	0.9638	0.0832	0.9238	**0.0499**
0.8128	0.3118	0.9151	0.2794	0.9063	0.3241	0.8813	**0.2683**
0.9726	0.165	0.9685	0.1486	0.9454	0.1939	0.9754	**0.0912**
0.9009	0.1065	0.9357	**0.0921**	0.9388	0.1135	0.8988	0.0989
0.9895	0.1777	0.994	0.1649	0.992	0.2053	0.9882	**0.1541**
0.989	0.1263	0.985	0.1273	0.9745	0.0949	0.9275	**0.0928**
0.9644	0.0878	0.9728	0.0995	0.9637	0.1042	0.9644	**0.0801**
0.9662	0.0579	0.9699	0.0646	0.9647	0.0705	0.9641	**0.0508**
0.9793	0.2022	0.7551	0.1755	0.7528	0.23	0.8328	**0.0963**
0.9938	0.0639	0.9395	0.0785	0.9359	0.076	0.9959	**0.0531**
0.9857	0.0628	0.9906	0.0643	0.9874	0.0693	0.9751	**0.0306**
0.9001	0.0627	0.9335	0.0708	0.8903	0.0694	0.9003	**0.0535**
0.9714	0.0445	0.8289	0.0501	0.8141	0.0494	0.9541	**0.0324**
0.9936	0.0897	0.9985	0.1050	0.9952	0.1078	0.9952	**0.0804**
0.9578	0.0753	0.9596	0.0703	0.9691	0.0894	0.9990	**0.0382**
0.9911	0.0363	0.9925	0.0409	0.9893	0.0429	0.9589	**0.0312**
0.9888	0.0438	0.9948	0.0495	0.9894	0.0462	0.9899	**0.0213**
0.7673	0.0446	0.8534	0.0525	0.8515	0.0574	0.8001	**0.0206**
0.9329	0.0267	0.9448	0.0243	0.9451	0.0283	0.9479	**0.0184**

Bold indicates the best achieved score

## Footnotes

1 webcam with image resolution 800 × 600.

2 Kinect for XBox 360: http://www.xbox.com/kinect Microsoft.

3 http://pics.psych.stir.ac.uk/.

4 http://cswww.essex.ac.uk/mv/allfaces/.

5 BSL Signbank, http://bslsignbank.ucl.ac.uk/.

6 http://www2.imm.dtu.dk/~aam/.

7 http://pics.psych.stir.ac.uk/.
